# Immunoglobulin Fc Heterodimer Platform Technology: From Design to Applications in Therapeutic Antibodies and Proteins

**DOI:** 10.3389/fimmu.2016.00394

**Published:** 2016-10-06

**Authors:** Ji-Hee Ha, Jung-Eun Kim, Yong-Sung Kim

**Affiliations:** ^1^Department of Molecular Science and Technology, Ajou University, Suwon, Korea; ^2^Department of Applied Chemistry and Biological Engineering, College of Engineering, Ajou University, Suwon, Korea

**Keywords:** bispecific antibody, Fc engineering, heterodimeric Fc, Fc-fusion proteins, immunocytokines, antibody engineering

## Abstract

The monospecific and bivalent characteristics of naturally occurring immunoglobulin G (IgG) antibodies depend on homodimerization of the fragment crystallizable (Fc) regions of two identical heavy chains (HCs) and the subsequent assembly of two identical light chains (LCs) via disulfide linkages between each HC and LC. Immunoglobulin Fc heterodimers have been engineered through modifications to the CH3 domain interface, with different mutations on each domain such that the engineered Fc fragments, carrying the CH3 variant pair, preferentially form heterodimers rather than homodimers. Many research groups have adopted different strategies to generate Fc heterodimers, with the goal of high heterodimerization yield, while retaining biophysical and biological properties of the wild-type Fc. Based on their ability to enforce heterodimerization between the two different HCs, the established Fc heterodimers have been extensively exploited as a scaffold to generate bispecific antibodies (bsAbs) in full-length IgG and IgG-like formats. These have many of the favorable properties of natural IgG antibodies, such as high stability, long serum half-life, low immunogenicity, and immune effector functions. As of July 2016, more than seven heterodimeric Fc-based IgG-format bsAbs are being evaluated in clinical trials. In addition to bsAbs, heterodimeric Fc technology is very promising for the generation of Fc-fused proteins and peptides, as well as cytokines (immunocytokines), which can present the fusion partners in the natural monomeric or heterodimeric form rather than the artificial homodimeric form with wild-type Fc. Here, we present relevant concepts and strategies for the generation of heterodimeric Fc proteins, and their application in the development of bsAbs in diverse formats for optimal biological activity. In addition, we describe wild-type Fc-fused monomeric and heterodimeric proteins, along with the difficulties associated with their preparations, and discuss the use of heterodimeric Fc as an alternative scaffold of wild-type Fc for naturally monomeric or heterodimeric proteins, to create Fc-fusion proteins with novel therapeutic modality.

## Introduction

Bispecific antibodies (bsAbs) simultaneously bind two different antigens or two distinct epitopes on the same antigen as a single molecule; they differ from naturally occurring immunoglobulin G (IgG) monospecific antibodies (mAbs) (1, 2). Owing to their additional targeting ability, bsAbs often offer improved clinical benefits for the treatment of complicated diseases, such as cancers and immune disorders, wherein multiple cell-surface receptors or ligands are engaged (1–3). Numerous efforts have been made to engineer mAbs into bsAbs, which has resulted in the generation of more than 60 different bsAb formats (3–5). Many bsAbs have been engineered by linking antibody fragments, such as single-chain variable fragments (scFv), antigen-binding fragments (Fab), and heavy (VH) and light chain (VL) variable domains, as well as their appendages to IgG-format mAbs (3–6). However, these novel formats, deviating from the conventional IgG structure, often suffer from poor physicochemical properties, such as low solubility and aggregation, difficulties in large-scale manufacturing, poor pharmacokinetics, and potential immunogenicity (3–6). To improve the developability, bsAbs in the formats of intact IgG or IgG-like (containing an Fc) architectures have been extensively developed (5–7).

Conventional IgG antibodies are bivalent and monospecific, the assembly of which depends upon *in vivo* homodimerization of two identical heavy chains (HCs), which is mediated by homodimeric associations between CH3 domains, and subsequently disulfide linkages between each HC and each light chain (LC), in B cells (8–11). Thus, the development of bsAbs, using intact IgG formats with wild-type HCs and LCs, faces HC–HC and HC_VH-CH1_–LC mispairing problems (5, 7). Some approaches for IgG-based bsAbs, based on wild-type homodimeric Fc regions, have been utilized, such as a dual-action Fab (DAF, two-in-one antibody) (12), a κλ-body (13), and a rat/mouse chimeric antibody (14). However, DAF and κλ-body technologies require extensive antibody engineering and screening and are not easy to generate with previously established mAbs. The rat/mouse chimeric antibody requires multiple purification stages, suffers from low purification yields, and faces potential immunogenicity.

To address the HC mispairing problem, heterodimeric Fc technology, which enables two different HCs to be preferentially assembled together, rather than with the same HCs, has been developed (5, 7, 15). Heterodimeric, Fc-based, intact IgG-format bsAbs have been developed in combination with a common LC approach (16) or with two distinct LCs, using the CrossMab technology (17) and ortho-Fab IgG technology (18). Additional heterodimeric Fc scaffolds have been extensively exploited for the generation IgG-like bsAbs by appending antigen-binding antibody fragments, such as VH, VL, scFv, Fab, and single-chain Fab (scFab). Depending on the designed architectures, the resulting bsAbs differ in antigen specificity (from monospecific to tetraspecific) and antigen-binding valency (from monovalent to tetravalent). In addition to the heterodimeric IgG scaffolds for bsAbs, heterodimeric Fc fragments are now emerging as excellent scaffolds to create Fc-fused monomeric or heterodimeric proteins or cytokines, which are challengeable formats to be achieved by wild-type homodimeric Fc.

In this review, we first focus on the design and generation of heterodimeric Fc and their application in the development of therapeutic bsAbs in diverse formats. We then describe the current status of homodimeric Fc-fused monomeric proteins and peptides and propose that heterodimeric Fc fragments, which can present the fusion partner as native-like monomeric or heterodimeric forms, represent a promising scaffold for the next generation of Fc-fused proteins and cytokines.

## Heterodimeric Fc Engineering

**Figure 1:**
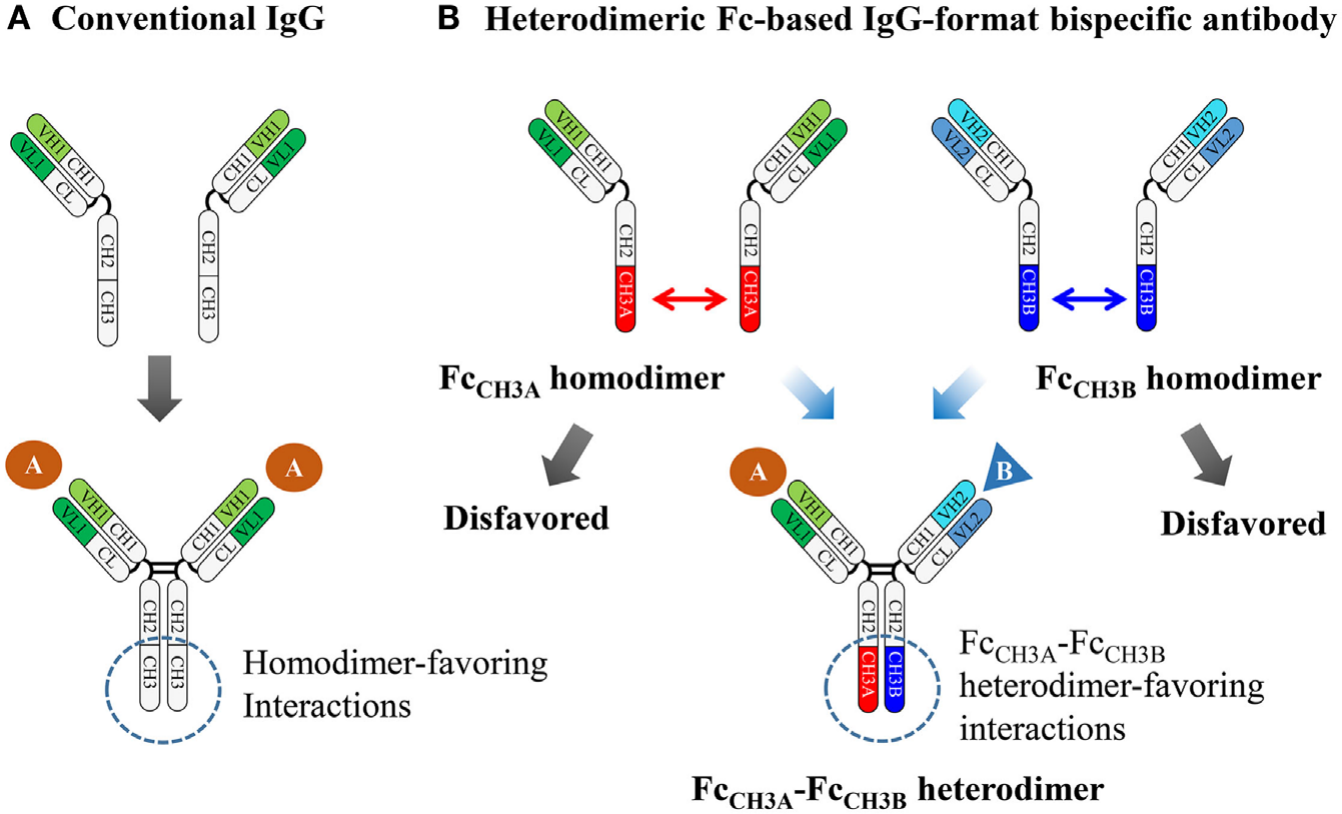
**Schematic diagram showing the assembly of conventional IgG and heterodimeric Fc-based IgG-format bsAbs**. **(A)** The homodimeric interactions between the wild-type CH3 domains are the initial driving force for HC homodimerization and subsequently disulfide bonds in the hinge regions and between the HC and LC complete the assembly of conventional IgGs, which are bivalent and monospecific. **(B)** Heterodimer Fc technology introduces asymmetric mutations in each CH3 domain, which enforces two different HCs to be predominantly assembled together, while disfavoring homodimerization between the same HCs. Heterodimeric Fc fragments facilitate the generation of IgG-format bsAbs, which can simultaneously bind to two different antigens.

Wild-type Fc homodimerization is initially mediated by a large, tightly packed interface (~2469 Å^2^ buried surface area), between the two identical CH3 domains with sub-nanomolar affinity, and subsequently by disulfide linkages in the hinge region (Figure 1A) (8, 9). For this reason, heterodimeric Fc variants have been mainly engineered through the replacement of homodimer-favoring interactions at the CH3 domain interface with heterodimer-favoring interactions. This is achieved by introducing asymmetric mutations in each CH3 domain, which promotes the assembly of HCs from two different antibodies (Figure 1B). This heterodimeric Fc engineering, using CH3 variant pairs, has been approached using two strategies: (1) structure-based rational design and (2) directed evolution.

**Figure 2:**
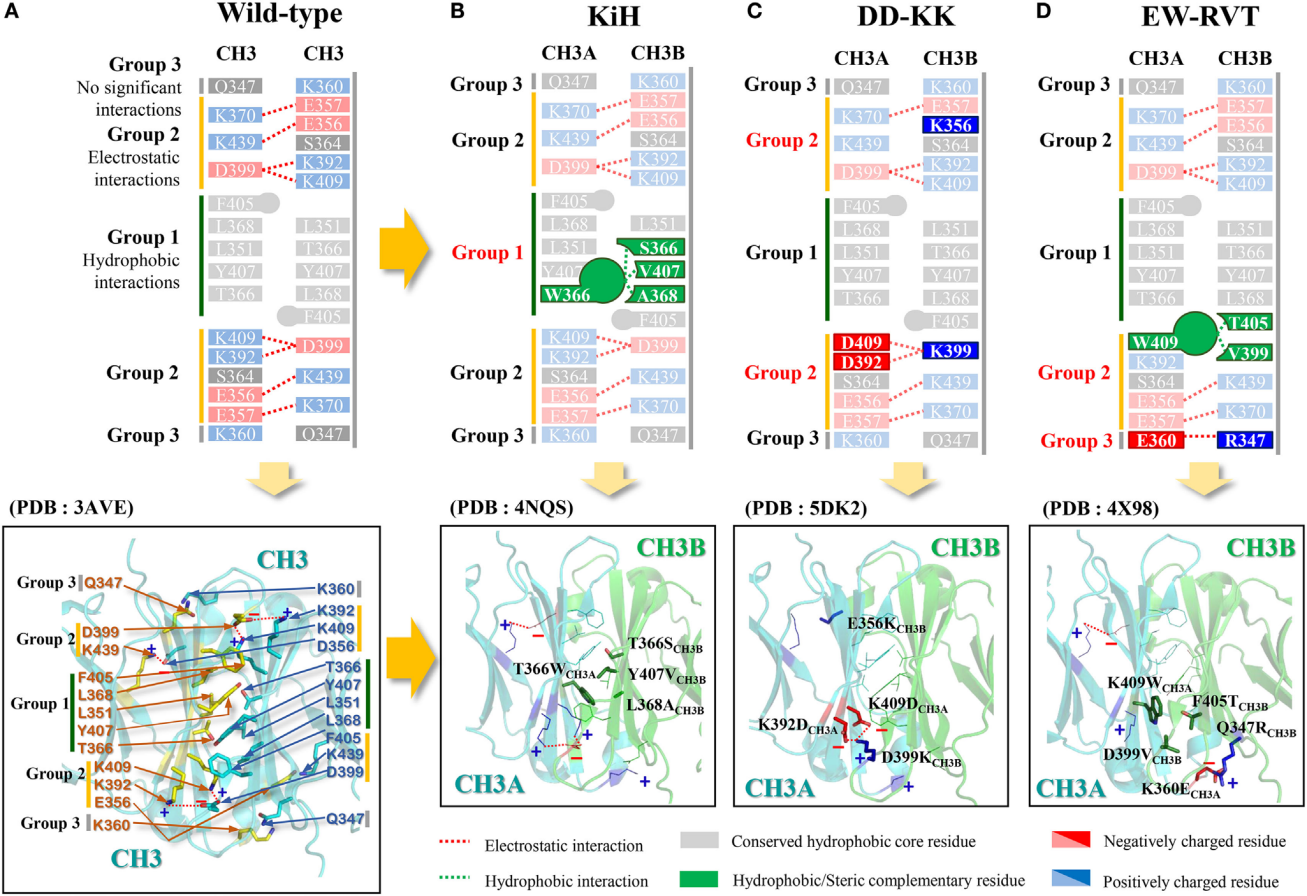
**Schematics of the major interactions contributing to homodimeric CH3 interactions in the wild-type (A) and heterodimeric CH3A–CH3B interactions in the KiH (B), DD-KK (C), and EW-RVT (D) heterodimeric Fc variants**. In **(A)**, due to the twofold symmetry of the inter-CH3 interface, each pairwise interaction is represented twice in the structure. In **(B–D)**, the upper panels highlight the main heterodimeric Fc-driving interactions with the indicated color codes. The lower panels show the crystal structures of the wild-type Fc [PDB 3AVE (19)] and the representative heterodimeric Fc variants of KiH [PDB 4NQS (28)], DD-KK [PDB 5DK2 (29)], and EW-RVT [PDB 4X98 (30)], highlighting the inter-CH3 domain interfaces. The images were generated using PyMol software (DeLano Scientific LLC).

X-ray crystal structures of human IgG1 Fc show that Fc homodimerization is driven by both hydrophobic interactions at the center of the CH3 interface core and symmetric electrostatic interactions surrounding the rim of the hydrophobic core (19, 20), as schematically shown in Figure 2A. Thus, structure-based rational design of heterodimeric Fc fragments has been utilized to generate heterodimeric CH3 variant pairs (CH3A:CH3B) with different mutations in each chain at the CH3 interface core such that the variant pair thermodynamically favors the formation of heterodimers over the homodimers. The structure-based rational design of such heterodimeric CH3 variant pairs can be classified into four strategies: (i) symmetric-to-asymmetric steric complementarity design (e.g., KiH, HA-TF, and ZW1) (21–24), (ii) charge-to-charge swap (e.g., DD-KK) (25), (iii) charge-to-steric complementarity swap plus additional long-range electrostatic interactions (e.g., EW-RVT) (26), and (iv) isotype strand swap [e.g., strand-exchange engineered domain (SEED)] (7, 24), as summarized in Table 1. Critical parameters in heterodimeric Fc engineering include the yield of heterodimeric Fc formation over unwanted homodimeric Fc contaminants, minimal loss in stability relative to the natural Fc, and maintaining natural Fc-like properties, such as serum half-lives and effector functions. Heterodimeric Fc-directed bsAb formation usually yields greater than 90% of the desired product by co-expressing two heterodimer Fc-based antibodies. This makes it feasible for large-scale production and quality control to meet clinical needs. In contrast to heterodimeric Fc engineering, an approach to generate monomeric Fc fragments was explored by introducing four mutations at the CH3 interface (27).

**Table 1:** **Design and strategies for heterodimeric Fc variants**.

Heterodimeric Fc name	Paired mutations		Heterodimer-favoring interactions	PDB ID	Reference
	CH3A chain	CH3B chain			
KiH	T366W	T366S/L368A/Y407V	Hydrophobic/steric complementarity	4NQS/5DI8/5HY9	(28)
KiH_S-S_	T366W/S354C	T366S/L368A/Y407V/Y349C	KiH + inter-CH3 domain S-S bond	–	(16, 29)
HA-TF	S364H/F405A	Y349T/T394F	Hydrophobic/steric complementarity	–	(23)
ZW1	T350V/L351Y/F405A/Y407V	T350V/T366L/K392L/T394W	Hydrophobic/steric complementarity	4BSW	(24)
7.8.60	K360D/D399M/Y407A	E345R/Q347R/T366V/K409V	Hydrophobic/steric complementarity + electrostatic complementarity	5DJZ (Partial)	(29)
DD-KK	K409D/K392D	D399K/E356K	Electrostatic complementarity	5DK2	(25)
EW-RVT	K360E/K409W	Q347R/D399V/F405T	Hydrophobic/steric complementarity plus long-range electrostatic interaction	4X98	(26, 30)
EW-RVT_S-S_	K360E/K409W/Y349C	Q347R/D399V/F405T/S354C	EW-RVT + inter-CH3 domain S-S bond	4X99	(30)
SEED	IgA-derived 45 residues on IgG1 CH3	IgG1-derived 57 residues on IgA CH3	Hydrophobic/steric complementarity (strand exchange between IgG and IgA)	–	(31)
A107	K370E/K409W	E357N/D399V/F405T	Hydrophobic/steric complementarity + hydrogen bonding complementarity (directed evolution using yeast surface display)	–	(32)

### Symmetric-to-Asymmetric Steric Complementarity Design

In a pioneering approach, Carter and colleagues from Genentech conceived the heterodimeric Fc variant and invented the so-called “Knobs-into-Holes (KiH)” Fc technology (21, 22). The concept was to introduce a “knob” in one CH3 domain (CH3A) by substitution of a small residue with a bulky one (i.e., T366W_CH3A_ in EU numbering). To accommodate the “knob,” a complementary “hole” surface was created on the other CH3 domain (CH3B) by replacing the closest neighboring residues to the knob with smaller ones (i.e., T366S/L368A/Y407V_CH3B_) (Figure 2B) (15). The “hole” mutation was optimized by structured-guided phage library screening (21). X-ray crystal structures of KiH Fc variants (28, 33) demonstrated that heterodimerization is thermodynamically favored by hydrophobic interactions driven by steric complementarity at the inter-CH3 domain core interface, whereas the knob–knob and the hole–hole interfaces do not favor homodimerization owing to steric hindrance and disruption of the favorable interactions, respectively. The KiH Fc was further engineered to improve purity and stability by introducing an additional inter-CH3 domain disulfide bond pair, S354C_CH3A_–Y349C_CH3B_, generating the KiH_S-S_ Fc variant. This exhibited a high yield of heterodimerization (~95%) and improved thermal melting (*T*_m_) of the CH3 domain by 6 to ~78°C (16). Most KiH Fc-based bsAbs currently being evaluated in clinical trials have adopted the KiH_S-S_ Fc as a scaffold (Table 2).

**Table 2:** **Heterodimeric Fc-based antibody formats, generated in previous studies, and now being evaluated in clinical trials**.

Antibody format	Heterodimeric Fc scaffold	Fusion format (target)		Clinical trials	Reference
		Fc_CH3A_	Fc_CH3B_		
**Monospecific and monovalent antibodies**					
VH-Fc/VL-Fc	EW-RVT	VH (c-Met)	VL (c-Met)	–	(26)
Fc/Fab-Fc	KiH	–	Fab (c-Met)	Phase 3 (failed)	(34)
HC_VH-CH1_-Fc/LC-Fc	DD-KK	HC_VH-CH1_ (mTNFR1)	LC (mTNFR1)	–	(25)
**IgG-like formats with appendages of scFv and scFab**					
scFv-Fc/scFv-Fc	EW-RVT	scFv (c-Met)	scFv (VEGFR-2)	–	(26)
scFv-Fc/scFv-Fc	DD-KK	scFv (CD3)	scFv (TARTK)	–	(25)
Fc/scFv_2_-Fc (Fc/BiTE-Fc)	KiH	–	scFv_2_ (CD3/EpCAM)	–	(35)
Fab-Fc/scFv-Fc	HA-TF	Fab (CD123)	scFv (CD3)	Phase 1	(36)
		Fab (CD20)	scFv (CD3)	Phase 1	
Fab-Fc/scFab-Fc (OAscFab-IgG)	KiH_S-S_	Fab (EGFR)	scFab (IGF-1R)	–	(37)
scFab-Fc/scFab-Fc-scFv	KiH_S-S_	scFab-Fc (EGFR)	scFab-Fc-scFv (IGF1R/HER3)	–	(38)
scFab-Fc-scFv/scFab-Fc-scFv	KiH_S-S_	scFab-Fc-scFv (EGFR/HER3)	scFab-Fc-scFv (IGF1R/HER3)	–	(38)
Fab-CrossMab^CH1-CL^ IgG	KiH_S-S_	Fab (CEA)	Fab-CrossFab^CH1-CL^ (CEA/CD3)	Phase 1	(39, 40)
Fv-/Fv-CrossMab^CH1-CL^ IgG	KiH_S-S_	Fv-Fab-Fc (HER3/VEGF)	Fv-CrossFab^CH1-CL^-Fc (HER2/EGFR)	–	(41)
**Intact IgG formats with correct LC association**					
Common LC-IgG	KiH_S-S_	Fab (FIXa)	Fab (FX)	Phase 3	(42)
CrossMab^CH1-CL^ IgG	KiH_S-S_	Fab (Ang-2)	CrossFab^CH1-CL^ (VEGFA)	Phase 1 (RG7221); Phase 2 (RG7716)	(17, 39)
Four-in-one CrossMab^CH1-CL^ IgG	KiH_S-S_	Dual-action Fab (EGFR/HER3)	Dual-action CrossFab^CH1-CL^ (HER2/VEGF)	–	(41)
Ortho-Fab IgG	DD-KK	Ortho-Fab (EGFR)	Ortho-Fab (c-Met)	Phase 1	(18)
**Intact IgG formats with *in vitro* assembly**					
IgG	KiH	Fab (BACE1)	Fab (TfR)	–	(43, 44)

Extensive strategies combining structure-based computational design of many variants and subsequent experimental validation to assess the heterodimerization yield have been adopted to generate other heterodimeric Fc variants with sterically complementary mutations. These include HA-TF Fc from Xencor (23) and ZW1 Fc from Zymeworks (24). Particularly, ZW1 was generated by a two-stage approach, combining negative design to first destabilize the natural Fc homodimer-favoring interactions at the CH3–CH3 interfaces and then positive design of the previously designed variant to increase biophysical stability (24, 45). Negative design, maximizing unfavorable interactions in potential homodimers while promoting specificity between heterodimeric species, generated an Fc variant with F405A/Y407V_CH3A_–T366L/T394W_CH3B_, and resulting in a heterodimerization yield of ~95% (24). However, the *T*_m_ of the CH3 domain was ~72°C, similar to that of KiH Fc (22), but ~10°C lower than that of the wild-type CH3 domain (30). The subsequent positive design, which added T350V/L351Y_CH3A_–T350V/K392L_CH3B_ mutations to the first variant, yielded ZW1 (T350V/L351Y/F405A/Y407V_CH3A_–T350V/T366L/K392L/T394W_CH3B_), which retained the high Fc heterodimerization yield of ~95% and exhibited Fc stability similar to that of the wild-type CH3 domain with a *T*_m_ value of 81.5°C (24). The high heterodimer purity of ZW1, validated by stable expression in Chinese hamster ovary (CHO) cells, and the favorable biophysical properties could improve the manufacturability of ZW1-based bsAbs (45).

More recently, Leaver-Fay et al. (29) computationally produced heterodimeric Fc variants, using an explicit multistate design (sequence optimization) combined with negative design to destabilize and eliminate homodimer-favoring inter-CH3 interactions. One of the best clones, 7.8.60 Fc, was first designed by introducing mutations of D399M_CH3A_–K409V_CH3B_ to eliminate the conserved electrostatic interactions of D399-K392/K409 in the wild-type CH3 interface. In the next-round, computational optimization identified the following substitutions sequentially: Y407A_CH3A_–T366V_CH3B_ to minimize CH3B homodimerization and to improve the heterodimer formation, K360D_CH3A_ − Q347R_CH3B_ to eliminate CH3B homodimers, and finally E345R_CH3B_ to strengthen the K360D_CH3A_−Q347R_CH3B_ interaction (Table 1). The crystal structure determined clearly resolved the interface and showed that the models were very accurate. The 7.8.60 heterodimeric Fc combined with orthogonal Fab interface mutation technology (18) produced intact IgG-format bsAbs with ~93% purity (29).

### Charge-to-Charge Swap Design

As an alternative approach, Gunasekaran et al. from Amgen (25) sought to design a heterodimeric Fc by reversing symmetric charge complementarity at the CH3 domain interface, while retaining hydrophobic core integrity (Figure 2C). Owing to twofold symmetry at the inter-CH3 interface, the targeted electrostatic interaction pairs were symmetrically duplicated on both peripheral sides of the hydrophobic core (Figure 2A). They converted the symmetric charge-pair residues into asymmetric charge polarity by first designing a K409D_CH3A_–D399K_CH3B_ pair variant and then further screening for additional charge-pair mutations through experimentally assessing heterodimerization yield. This resulted in DD-KK with K409D/K392D_CH3A_–D399K/E356K_CH3B_ mutation pairs. The asymmetric charge mutation pairs drove heterodimerization, whereas “positively charged” and “negatively charged” homodimers were suppressed by unfavorable repulsive charge interactions. Particularly, E356K_CH3B_ was introduced in an attempt to suppress the Fc_CH3B_–Fc_CH3B_ homodimerization to increase the purity of Fc_CH3A_–Fc_CH3B_ heterodimer. The DD-KK design resulted in greater than 90% heterodimerization depending on the transfection ratio of the two chains.

### Charge-to-Steric Complementarity Swap and Long-Range Electrostatic Interaction Design

The concept of KiH and DD-KK Fc design can be summarized as the replacement of the symmetric hydrophobic and electrostatic interactions conserved at the CH3 interface with the same, but asymmetric, interactions. In contrast to the sterics-based KiH design and charge-swap DD-KK design, Choi et al. (26) replaced the conserved, symmetric electrostatic interactions at the buried interface of the CH3 domains with asymmetric hydrophobic interactions. This resulted in the generation of a W-VT Fc variant with K409W_CH3A_–D399V/F405T_CH3B_ mutations, favoring Fc heterodimer formation (~77% purity) due to complementary hydrophobic interactions, while disfavoring homodimers owing to the loss of the K409–D399 electrostatic interactions and the steric collision of K409W_CH3A_–F405_CH3A_. To increase the heterodimerization yield of W-VT, they replaced a K360–Q347 pair with negligible interactions, owing to the long distance (4.61 Å) at the rim of the CH3 interface (19) with the K360E_CH3A_–Q347R_CH3B_ asymmetric mutation pair, which favors the heterodimer because of the long-range electrostatic interaction at a distance of 3.45 Å (30). The combined heterodimeric Fc, called EW-RVT (Figure 2D), showed a heterodimer yield of ~91% and a CH3 domain *T*_m_ value of ~77.5°C, which were comparable to those of KiH and DD-KK Fc variants (26). The X-ray crystal structure of the EW-RVT Fc heterodimer led to the addition of an inter-CH3 disulfide bond with a Y349C_CH3A_–S354C_CH3B_ pair, yielding EW-RVT_S-S_ Fc. The asymmetric disulfide bond was confirmed by resolving the crystal structure (30). EW-RVT_S-S_ Fc showed improved heterodimer yield (by ~3%) and higher thermodynamic stability of the CH3 domain (by ~2.8°C) compared to those parameters of the parent EW-RVT Fc (30). The crystal structures of EW-RVT and EW-RVT_S-S_ Fc fragments, obtained to determine the molecular details of the CH3A/CH3B interface, revealed that the mutations did not cause any significant changes in the Fc structure (30). In agreement with the crystal structure, the EW-RVT Fc heterodimer displayed the native IgG1-like, pH-dependent, neonatal Fc receptor (FcRn) binding pattern, and Fcγ receptor (FcγR) interaction, which were equivalent to those of the native IgG1 (previously reported) (46, 47). This suggests that the EW-RVT-based antibody will have similar serum half-lives and effector functions to those of the conventional human IgG1.

### Isotype Strand Swap Design

Davis et al. from Merck Serono (31) designed a heterodimeric Fc by mixing human IgG and IgA CH3 domain segments to create a complementary CH3 heterodimer, which was referred to as SEED Fc. Native CH3 domains of human IgA and IgG are structurally similar (10), but cannot dimerize because of lower homology within interface residues. They analyzed the structural dimerization motif in the CH3 domain of each isotype and devised SEED through the exchange of some β-strand segments in the CH3 domain of each isotype to drive heterodimer formation through steric complementary contact surfaces. The SEED-based antibodies (e.g., SEEDbody) were purified with a protein A resin and exhibited long serum half-life and effector functions comparable to those of wild-type human IgG1 Fc-based antibodies (48). However, owing to the artificial sequence of the SEED CH3 heterodimer, the potential immunogenicity has not been determined.

### Directed Evolution of Heterodimeric Fc

Aforementioned Fc heterodimers have been generated through structure-based rational design or computational modeling. Recently, Choi et al. (32) generated heterodimeric Fc variants through directed evolution combined with yeast surface display and high-throughput screening (49, 50). A combinatorial heterodimeric Fc library display system was developed by mating two haploid yeast cell lines; one haploid cell line displayed an Fc chain library (displayed Fc_CH3A_) with mutations in one CH3 domain on the yeast cell surface, and the other cell line secreted an Fc chain library (secreted Fc_CH3B_) with mutations in the other CH3 domain. In the mated cells, secreted Fc_CH3B_ was displayed on the cell surface through heterodimerization with the displayed Fc_CH3A_. Fluorescence-based detection of this interaction enabled screening of the library for heterodimeric Fc variants by flow cytometry. For the proof-of-concept, they constructed combinatorial heterodimeric Fc libraries with simultaneous mutations on targeted residues of both CH3A and CH3B based on the template of the W-VT Fc variant, and screened the libraries to isolate numerous heterodimeric Fc variants with heterodimerization yields of ~90%. This directed evolution approach identified unexpected heterodimer-favoring mutation pairs at the CH3 interface, such as hydrogen bonding or cation–π interactions, as well as homodimer-disfavoring pairs, which have not been tested by structure-guided rational design. The best clone, A107, exhibited a heterodimerization yield of ~93%, which was much higher than that (~77%) of the parent W-VT Fc variant.

## Heterodimeric Fc-Based Antibodies in Diverse Formats

**Figure 3:**
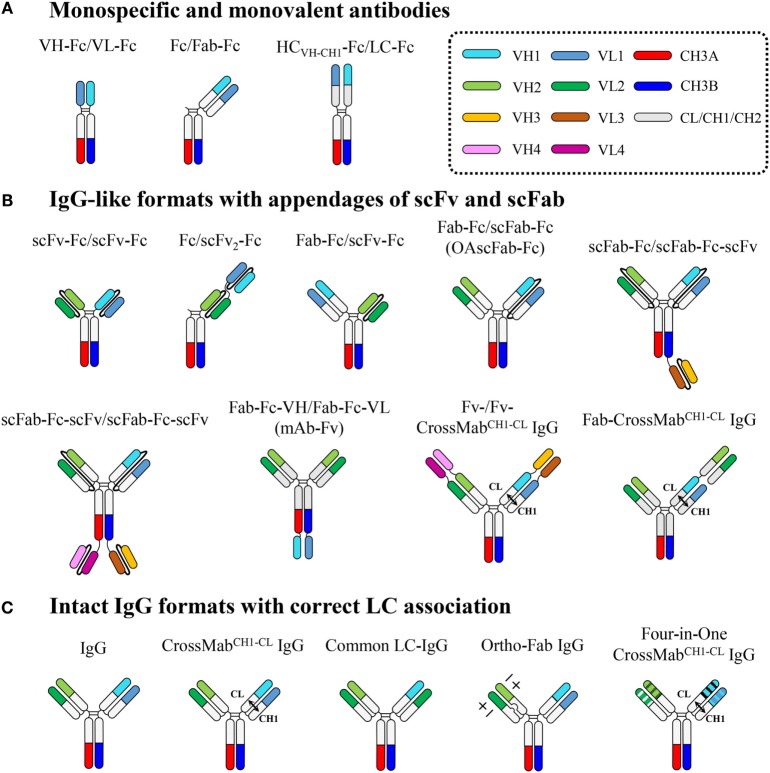
**Overview of heterodimeric Fc-based antibodies, which are subdivided into three classes: (A) monospecific and monovalent antibodies, (B) IgG-like formats with appendages of scFv and scFab, and (C) intact IgG formats with correct LC association**. Each domain is color coded as indicated in the dotted box. Connecting peptide linkers in scFv and scFab fragments are shown by thin black lines. Details are described in the text.

Heterodimeric Fc are compatible with independent fusion of two different antigen-binding units to the N- and/or C-terminus of each Fc chain. This facilitates the creation of diverse heterodimeric Fc-based antibodies with differences in specificity, binding valency, geometry of target-binding sites, among others, as summarized in Figure 3 and Table 2.

### Monospecific and Monovalent Antibodies

Heterodimeric Fc-based monospecific antibodies (mAbs) with monovalent antigen binding have been generated by fusion of the Fab to the N-terminus of only one Fc chain (Fc/Fab-Fc), the scFv to the N-terminus of only one Fc chain (Fc/scFv-Fc), the VH-CH1 (HC_VH-CH1_) and VL-CL to the respective N-termini of each heterodimeric Fc chain (HC_VH-CH1_-Fc/LC-Fc), or the VH and VL to the respective N-termini of each heterodimeric Fc chain (VH-Fc/VL-Fc) (Figure 3).

The Y-shape of typical IgG antibodies often limits their utility against some targets, whereby bivalent target-binding dimerizes and agonizes, rather than antagonizes, the intended targets. For example, targeting the c-Met receptor tyrosine kinase with bivalent antibodies can mimic the ligand hepatocyte growth factor (HGF)-mediated agonism via receptor dimerization (51). To address this issue, Genentech developed a KiH Fc-based, one-armed Fc/Fab-Fc format monovalent antibody, onartuzumab (MetMab), through fusion of a humanized anti-c-Met 5D5 Fab to the N-terminus of only the “hole” of the Fc chain (34). Whereas the bivalent anti-c-Met 5D5 IgG antibody acted as an agonist causing c-Met-activated tumor cell growth, rather than growth inhibition, the monovalent MetMab bound the receptor in a one-to-one fashion to inhibit HGF binding and block receptor activation, thus acting as an antagonist. Co-expression of three chains, specifically the Fc, HC_VH-CH1_-Fc, and LC, from three respective cistrons on a single plasmid, and targeting these chains to the periplasmic space of *Escherichia coli*, efficiently resulted in the production of MetMab. Purification by size-exclusion chromatography yielded ~95% purity (34). A clinical trial of MetMab combined with the epidermal growth factor receptor (EGFR) kinase inhibitor erlotinib to block metastasis in non-small cell lung cancer was recently halted in Phase 3, but other clinical trials with MetMab are expected. The EW-RVT Fc variant was also applied to make a MetMAb-like monovalent antibody in the VH-Fc/VL-Fc format, called msMet, wherein the VH and VL of MetMab were fused to the N-terminus of each heterodimeric Fc chain (26). As a single agent, the msMet antibody suppressed the *in vivo* tumor growth of human gastric cancer xenografts in mice.

DD-KK Fc was exploited to generate a monovalent 14D2 antibody, in the HC_VH-CH1_-Fc/LC-Fc format, to target tumor necrosis factor receptor 1 (TNFR1) by linking the VH-CH1 and VL-CL of the anti-TNFR1 14D2 antibody to the N-terminus of each heterodimeric Fc chain (25). The parent bivalent 14D2 antibody showed undesirable activity, specifically activation of TNFR1 at low doses via cross-linking of the receptor, similar to that observed for the TNF ligand. However, the monovalent antibody was devoid of agonistic activity, and blocked TNF-mediated chemokine induction in mice. Importantly, the monovalent 14D2 antibody exhibited similar pharmacokinetic profiles as the full-length IgG when injected in mice.

### IgG-Like Formats with Appendages of scFv and scFab

Heterodimeric Fc technology can resolve the HC mispairing problem, but still faces the challenge of a LC-pairing problem, specifically, cognate HC_VH-CH1_-LC pairing in the Fab region, to generate bsAbs in intact IgG formats (5, 7). To address the HC_VH-CH1_-LC-pairing problem, single-chain antigen-binding units, such as scFv and scFab, have been, respectively, linked to each heterodimeric Fc chain or tandemly linked to one heterodimeric Fc chain, generating IgG-like bsAbs in formats of scFv-Fc/scFv-Fc, scFab-Fc/scFab-Fc, Fc/scFv_2_-Fc, Fab-Fc-VH/Fab-Fc-VL, Fab-Fc/scFv-Fc, Fab-Fc/scFab-Fc, and scFab-Fc-(scFv)/scFab-Fc-scFv (Figure 3).

The EW-RVT Fc was exploited to generate a bsAb, dubbed bsVeMet, simultaneously targeting both c-Met and VEGF receptor 2 (VEGFR-2), with two respective antigen-specific scFvs fused to the N-termini of each heterodimeric Fc in the format of scFv-Fc/scFv-Fc (26). The bsVeMet more potently inhibited HGF and VEGF-stimulated cell proliferation, downstream signaling, and *in vivo* angiogenesis than the two parent mAbs.

Furthermore, bsAbs have been extensively designed to recruit immune effector cells, such as T cells, to tumor cells (52, 53). Bispecific T-cell Engager (BiTE) was constructed by tandem connection of two scFvs, one of which was specific to CD3 on T cells and the other was specific to a tumor-associated antigen on tumor cell surfaces. The goal of this strategy was to engage cytotoxic T cells for lysis of the targeted antigen-expressing cancer cells, and was first developed by Mack and colleagues (54). Later, the CD19 × CD3 BiTE blinatumomab was developed by Micromet and clinically approved in 2014 for the treatment of relapsed/refractory B-cell acute lymphocytic leukemia (55). However, the serum half-life of tandem scFv-based BiTEs in mice was only 5–6 h (56), which was much shorter than that of Fc-containing antibodies. The fast clearance of blinatumomab requires infusions or repeated injections to maintain a therapeutically effective dose over a prolonged period, specifically, daily infusions for 8 weeks in clinical trials (57). To improve pharmacokinetics of such scFv-based BiTEs, a DD-KK Fc-based bsAb was generated in the scFv-Fc/scFv-Fc format by fusing two scFvs with different binding specificities, one against human CD3 and the other against a tumor-associated receptor tyrosine kinase (TARTK), to each Fc heterodimer chain (25). Due to the presence of the Fc region, the scFv-Fc/scFv-Fc antibody exhibited a much longer *in vivo* half-life in mice and could be administered more infrequently compared to other types of CD3 bispecific scFv fragments (25). The CD3 × TARTK bsAb completely suppressed the growth of the TARTK-positive glioma in a xenograft mouse model.

Xu et al. from Sutro Biopharma (35) produced a KiH Fc-based BiTE bsAbs using the *E. coli*-based cell-free expression system. They designed epithelial cell adhesion molecule (EpCAM) × CD3 bsAbs in the two-armed scFv-Fc/scFv-Fc format or the one-armed Fc/scFv_2_-Fc (i.e., Fc/BiTE-Fc) format and further reversed the two formats by exchanging the “knob” Fc and “hole” Fc fusion partner. They found that the expression yield and biological activities, such as dual targeting and tumor cell killing by engaging T cells, was highly variable and depended on the formats. This suggested that a more sophisticated optimization process is necessary to determine the optimal ratio of the two expression plasmids for enhanced cell-free expression of the Fc fusion partner for the two scFvs or the tandem scFv BiTE. Intriguingly, the KiH Fc-based bsAb (100 kDa) showed significantly longer serum half-life (*T*_1/2_ ≈ 4.9–5.2 days) than the BiTE control (50 kDa) (T_1/2_ ≈ 0.2 days), but were less stable than trastuzumab IgG (150 kDa) (T_1/2_ ≈ 25 days) and scFv-Fc (100 kDa) (T_1/2_ ≈ 9.1 days) in mice. This might be attributed to destabilizing mutations in the CH3 domains of the KiH Fc (35).

Using an HA-TF Fc scaffold, Xencor generated Her2 × CD3 or Her2 × CD16 bsAbs in Fab-Fc-VH/Fab-Fc-VL (dubbed mAb-Fv) format through the fusion of VH and VL domains of CD3 or CD16 antibodies to the C-termini of two distinct HCs (23). The advantage of the mAb-Fv format is improved tumor targeting due to bivalent antigen engagement through two intact arms of the IgG mAb, compared to the monovalent target-binding formats, such as scFv-Fc/scFv-Fc and scFab-Fc/scFab-Fc. However, mAb-Fv showed non-native oligomeric species and the C-terminal Fv exhibited inferior affinity to the CD16 or CD3 antigen compared to the parent Fab. This was most likely due to steric hindrance and suggested the need for C-terminal-fused Fv optimization. For these reasons, Xencor adopted an HA-TF Fc-based Fab-Fc/scFv-Fc bsAb format (dubbed “plug-and-play” platform), which showed native mAb-like stability and non-compromised antigen-binding activity (36). For example, the Fab-Fc/scFv-Fc formatted bsAbs, such as CD123 × CD3 (XmAb14045) and CD20 × CD3 (XmAb13676), are expected to begin clinical Phase 1 trials for acute myeloid leukemia (NCT02730312) and B-cell malignancies, respectively, in 2016.^1^ Glenmark Pharm is also developing bsAbs in a heterodimeric Fc-based Fab-Fc/scFv-Fc format (dubbed “BEAT” by Glenmark), in which the heterodimeric Fc was designed by introducing mutations, mimicking the natural association of the heterodimeric T-cell receptor (TCR) α and β chains, into the interface between the two CH3 domains of IgG (58).

Schanzer et al. from Roche (37, 59) generated a KiH Fc-based bsAb XGFR in the format of Fab-Fc/scFab-Fc for targeting EGFR with the Fab format in one arm and the insulin-like growth factor receptor type I (IGF-1R) with scFab format in the other arm. The format of XGFR bsAb, the so-called one arm scFab IgG (OAscFab-IgG) was devised to prevent incorrect pairing of LCs, while maintaining the closest possible format to intact IgG. XGFR was produced with high expression yields comparable to those of the respective parent IgG mAbs. However, other tested bsAb formats of IgG-scFv (IGF-1R IgG with C-terminal attachment of disulfide-stabilized EGFR scFv) and dual-variable domain (DVD)-IgG (IGF-1R IgG with the N-terminal attachment of EGFR VH and VL in the HC and LC, respectively) showed ~2-fold and ~16-fold lower expression yields than that of the OAscFab-IgG format (37, 59). To further increase immune effector functions triggered by XGFR bsAb, the heterodimeric Fc portion was glycoengineered to remove the fucosylation, leading to strong antibody-dependent cell-mediated cytotoxicity. The resulting XGFR demonstrated potent anti-tumor efficacy in multiple mouse xenograft tumor models (37, 59). In addition, Castoldi et al. from Roche (38) constructed a KiH_S-S_ Fc-based trispecific antibody (dubbed TriMAb) in the scFab-Fc-(scFv)/scFab-Fc-scFv format, in which anti-EGFR and anti-IGF-1R scFabs are on the respective arm of IgG with anti-Her3 scFv to fused to the C-terminus of both chains or only one chain. The EGFR × IGF-1R × HER3 TriMAb showed growth inhibitory effects comparable to that of a combination of each single parental antibody, in a model cell proliferation assay.

### Intact IgG Formats with Correct LC Association

IgG-like bsAbs based on appending scFv and scFab to heterodimeric Fc fragments have overcome HC_VH-CH1_-LC mispairing issues within the Fab region, but they are limited in their use of linkers, the length and composition of which often needs to be optimized. A sub-optimal linker can cause undesirable problems, such as loss of antigen binding, due to binding site hindrance, poor expression levels, immunogenicity, poor pharmacokinetics, and *in vivo* cleavage. Thus, many attempts have been made to develop bsAbs that deviate minimally from natural IgG antibodies without the use of artificial linkers. Since antigen specificity usually resides in the Fv chain composed of the VH and VL, correct pairing between the cognate HC and LC during co-expression and assembly of bsAbs is essential to ensure antigen-binding specificity and affinity. LC mispairing occurs because the Fab heterodimerization interfaces of VH–VL and CH1–CL between two distinct antibodies are almost identical, though the complementarity determining regions (CDRs) of VH and VL are quite different. Thus, to generate bsAbs using a full-length IgG format, cognate HC_VH-CH1_-LC pairing should be ensured, in addition to correct HC pairing by heterodimeric Fc technology. The LC mispairing problem has been addressed by using a common LC (15, 16), a domain crossover (CrossMab) (17), and a designed orthogonal Fab interface (18) (Figure 3).

The use of a common LC, compatible with the two distinct HC_VH-CH1_s, is a straightforward way to construct bsAbs with intact IgG formats (15). Two mAbs can be first isolated from a human scFv phage library sharing identical VLs and then assembled into a single IgG format using KiH Fc-based HC heterodimerization (16, 60). However, this approach constrains antibody discovery for two different targets with a single LC or requires novel antibody libraries, and is difficult to adopt when using two different previously established antibodies. A notable example of an IgG-format bsAb using a common LC in combination with a KiH Fc is emicizumab (ACE910) developed by Chugai, which targets factor IXa and factor X (FIXa × FX) (61, 62). They first generated various FIXa- and FX-specific mAbs by immunizing mice with human FIXa and FX antigens and then generated a lead chimeric bsAb based on the retention of biological activity and the feasibility of using only one LC (62). FIXa and FX form a complex with cofactor FVIII but the complex formation is deficient in hemophilia A patients. Standard treatment for this disease includes frequent injection of recombinant or plasma-derived factor VIII (62). Emicizumab mimics FVIII functions by allowing FIXa and FX to be close for the formation of the complex. This is because the distance between the FIXa- and FX-binding sites of FVIIIa is similar to that between the two antigen-binding site arms of the Y-shaped IgG. Emicizumab overcomes the short half-life (T_1/2_ ≈ 0.5 days) of recombinant FVIII and is expected the lower immunogenicity. Emicizumab is now in a Phase 3 clinical study in patients with hemophilia A (NCT02622321) (42).

To facilitate antibody generation with a common LC, Merus developed a transgenic mouse (MeMo), engineered to generate antibodies with a single human common LC and diverse human HCs (63). The MeMo technology combined with their own CH3-domain engineered heterodimeric Fc technology allows them to generate intact IgG-format bsAb (dubbed Biclonics by Merus).^2^ A Biclonics-based bsAb MCLA-128 targeting Her2 × Her3 is now being evaluated in Phase 1/2 clinical trials for patients with solid tumors (64).

CrossMab technology from Roche (17) is another strategy to solve the HC_VH-CH1_–LC association problem in combination with KiH Fc technology. This technology keeps one Fab untouched, whereas the VH or CH1 domain of the other Fab is switched with partner VL or CL domains. In the early proof-of-concept stage of CrossMabs, they were evaluated in three formats according to the crossover region: (1) CrossMab^Fab^, crossover of the complete VH-CH1 and VL-CL domains, (2) CrossMab^VH-VL^, crossover of only the VH and VL domains, and (3) CrossMab^CH1-CL^, crossover of only the CH1 and CL domains. Although the three CrossMab variants showed comparable thermal stability to that of the parental IgG mAbs, the CrossMab^CH1-CL^ format showed only minimal unwanted side products; however, the other two formats produced some portion of side products (17). For this reason, the CrossMab^CH1-CL^ format was predominantly used over the other two formats. CrossMab-based bsAbs might be easily derived from pre-existing antibody pairs using domain crossover without the need for identification of common LC and the Fab interface mutations, required for correct LC association (39). Many CrossMAbs have been generated and evaluated and currently four different CrossMab-based bsAbs are now in active Phase 1/2 clinical trials (39) (Table 1). For example, two versions of a VEGF × Ang2 CrossMab^CH1-CL^ are currently being evaluated in clinical trials: (1) RG7221 (Vanucizumab), one VEGF × Ang2 CrossMab^CH1-CL^, in Phase 1 in patients with cancers (NCT01688206) and (2) RG7716, the other tailor-made VEGF × Ang2 CrossMab^CH1-CL^, in Phase 2 trials for patients with macular degeneration (NCT02484690).

The CrossMab^CH1-CL^ format was further extended by fusion of a Fab to the N-terminus to generate the Fab-CrossMab^CH1-CL^ format for trivalent bsAbs (Figure 3). Using this format, IgG-based T-cell bsAbs (TCBs) with bivalent binding to carcinoembryonic antigen (CEA), dubbed CEA-TCB, was developed (40). Currently, a Phase 1 clinical trial of CEA-TCB is ongoing (NCT02324257) for the treatment of CEA-expressing solid tumors (39). Furthermore, the genetic fusion of the two different DAFs (dual-action Fabs, i.e., two-in-one antibody), one EGFR × Her3 DAF and another Her2 × VEGF DAF, to the N-terminus of each arm of a KiH-based four-in-one CrossMab^CH1-CL^ format generated the EGFR × Her3 × Her2 × VEGF targeting tetraspecific IgG-format antibody (41) (Figure 3). The same group also generated an EGFR × Her3 × Her2 × VEGF targeting tetraspecific, tetravalent antibody by combining the CrossMab^CH1-CL^ and DVD-IgG technologies (65), resulting in the Fv-/Fv-CrossMab^CH1-CL^ IgG format (41) (Figure 3).

An alternative approach for enforcing correct HC_VH-CH1_–LC association includes introduction of a set of mutations at the heterodimeric VL–CL and VH–CH1 interface (18, 66, 67), similar to modification of the CH3 interface for the heterodimeric Fc design. In an ortho-Fab IgG approach (18), structure-based regional design introduced complementary mutations at the LC and HC_VH-CH1_ interface in only one Fab, without any changes being made to the other Fab (Figure 3). Based on the ortho-Fab IgG format combined with heterodimeric Fc technology, the EGFR × c-Met bsAb (LY3164530) from Eli Lilly was generated, which is currently being evaluated in a Phase 1 clinical study of patients with advanced or metastatic cancer (NCT02221882). Zymeworks is also currently developing intact IgG-format bsAbs generated by the combination of ortho-Fab IgG and ZW1 Fc technologies.^3^

### *In Vitro* Assembly of Intact IgG Formats

Heterodimeric Fc technology alone, without the combination of HC_VH-CH1_–LC association technology, can generate intact IgG-format bsAbs by separate expression of two parental half-antibodies [HC–LC (HL) pairs] and subsequent *in vitro* recombination into desirable bsAbs, the assembly of which is driven by heterodimer-favoring Fc interactions under controlled biochemical conditions (60). This approach allows the correct HC_VH-CH1_–LC association during the expression stage of the parental half-antibody and maintains the cognate HC_VH-CH1_–LC pairing in the next *in vitro* assembly step. Spiess et al. from Genentech (68) further combined the two separate processes into one step by co-culture of two transformed *E. coli* cell lines, each expressing a half-antibody. They demonstrated the robustness of the technology by producing 28 unique IgG bsAbs. However, this *E. coli* expression system was limited to production of non-glycosylated antibodies. Shatz et al. (69) applied this technology to a mammalian expression system to produce glycosylated antibodies. A notable example of a bsAb prepared using the above manufacturing system is the TfR × BACE1 bsAb that targets TfR (transferrin receptor) and BACE1 (β-site amyloid precursor protein-cleaving enzyme) (43, 44). In a human TfR knock-in mouse, the bsAb could cross the blood–brain barrier and accumulated in the mouse brain where it inhibited BACE1 to reduce brain Aβ production (44). This bsAb was validated in preclinical models, and is now on the path to clinical trials.

Unlike the human IgG1 isotype, IgG4 has been shown to undergo Fab arm exchange (FAE) both *in vivo* and *in vitro*, in which one half-antibody (HL pair) recombines with other half-antibodies from other IgG4 molecules (10, 70, 71). The structural basis of IgG4 for FAE resides in the IgG4 core hinge region (^226^Cys-Pro-Ser-Cys-Pro^230^), and especially the Ser228 residue and the CH3 domains (critically, the R409 residue) (70, 72). To confer FAE ability to IgG1, two groups engineered FAE-capable IgG1 by introducing the FAE-associated IgG4-specific mutation pairs at the core hinge region and inter-CH3 interface, for example, D221E/P228E/L368E_CH3A_–D221R/P228R/K409R_CH3B_ (dubbed EEE-RRR) from Rinat-Pfizer (73), or only at the inter-CH3 interface, such as K409R_CH3A_–K405L_CH3B_ (dubbed Duobody) from Genmab (72). The retention of the stable wild-type IgG1 hinge in the Duobody technology might be beneficial for the generation of bsAbs, which are resistant to reduction under physiological conditions *in vivo* and are much more stable during storage after FAE-mediated manufacturing (74). It should be noted that the above two mutation pairs at the CH3 interface are not enough to predominantly produce heterodimeric Fc fragments when the two HCs are co-expressed, unlike the KiH, DD-KK, and EW-RVT heterodimeric Fc technology. Instead, two mAbs based on the FAE-capable IgG1 are separately expressed and purified and then mixed together under mild redox conditions, resulting in a stable IgG1 bsAb with high yields (greater than 90%) (72–74). The EGFR × c-Met bsAb (JNJ-61186372) generated by Duobody technology was effective against EGFR inhibitor-resistant lung tumors in mice (75).

## Fc-Fused Proteins with the Natural Monomeric or Heterodimeric Form

**Figure 4:**
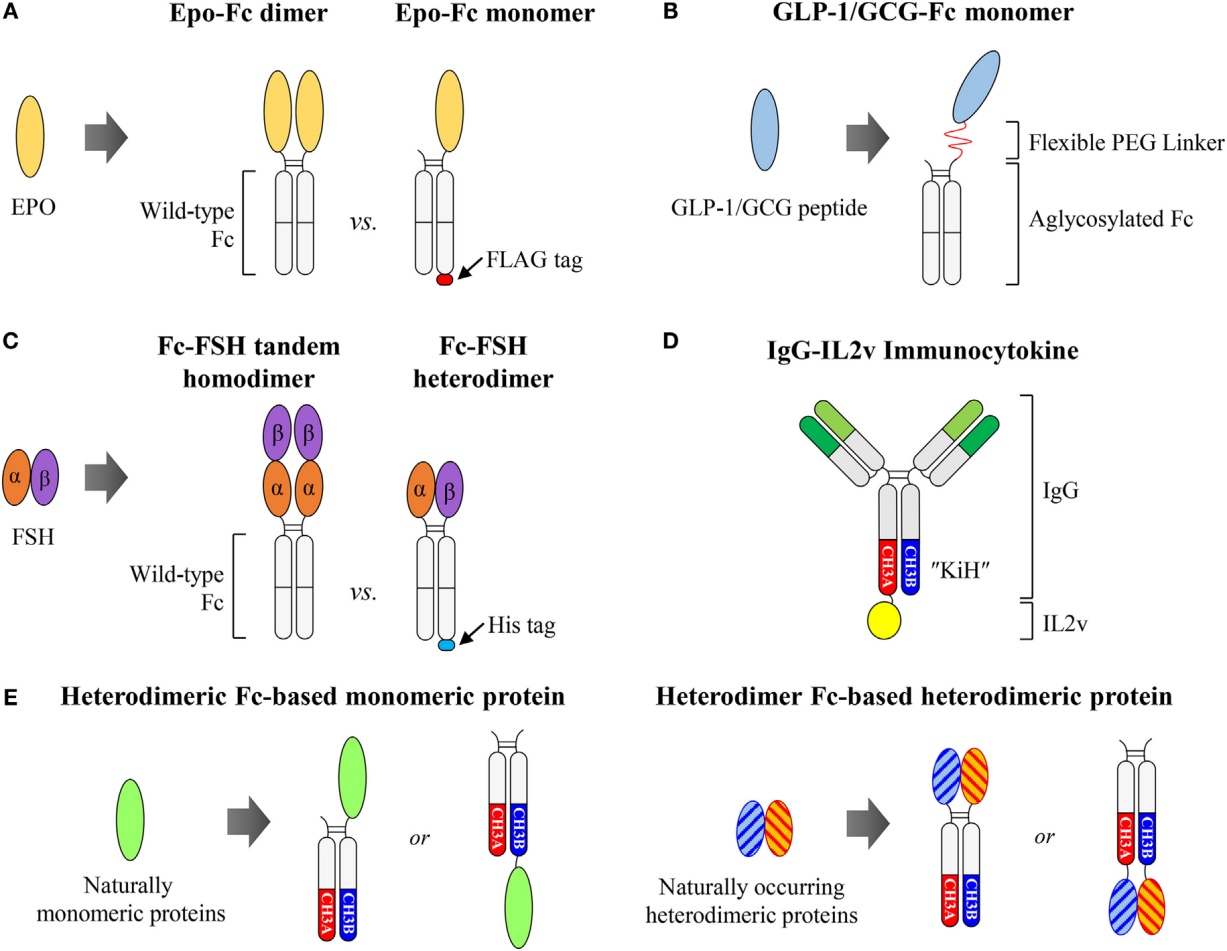
**Fc-fused monomeric or heterodimeric proteins**. **(A)** Wild-type Fc-based Epo-Fc dimer vs. Epo-Fc monomer. **(B)** Aglycosylated Fc-fused GLP-1/GCG monomeric peptide, generated by the LAPScovery technology. **(C)** Wild-type Fc-based Fc-FSH tandem homodimer vs. Fc-FSH heterodimer. **(D)** Heterodimeric Fc KiH-based IgG-fused IL2v monomer, IgG-IL2v, developed by Roche. **(E)** Potential use of heterodimeric Fc for the generation of Fc-fused monomeric or heterodimeric proteins to present the fusion partner in its naturally occurring form. The Fc-fused monomer can easily be generated by the fusion of monomeric protein to the N- or C-terminus of one heterodimeric Fc chain. The Fc-fused heterodimer can be generated by separate fusion of the two subunits of heterodimeric proteins to each chain of the heterodimeric Fc at the N- or C-terminus.

The genetic fusion of proteins, such as ligand-binding soluble receptors, cytokines, growth factors, enzymes, and peptides, to the Fc domain of human IgG1 is a well-established strategy to extend the serum half-life and often enhance biological activity through dimerization of the fusion partner (76–78). Conjugation to the Fc may also enhance tissue penetration of the fusion partner by FcRn-mediated transcytosis (79, 80). In addition, the Fc moiety in many cases improves the biophysical properties of its fusion partner, such as the solubility and stability of the protein (81). At the end of 2015, about 11 Fc-fusion proteins had been clinically approved (82). All of the marketed Fc-fusion proteins and many others in clinical trials are based on the wild-type human IgG1 Fc, which presents the fusion partner as a homodimer due to the homodimeric Fc structure. However, some cytokines and hormones naturally occurring in monomeric or heterodimeric forms exhibit markedly improved pharmacokinetics and even biological activities when they are fused to the Fc in natural forms (83). Thus, Fc-fusion, while keeping the original configuration of the fusion partners, is desirable for those proteins. However, as described below, generation of such Fc-fused monomeric or heterodimeric proteins based on the wild-type Fc is not straightforward (Figures 4A–C) due to the homodimeric structure. A promising solution to this limitation is the use of a heterodimeric Fc as an alternative scaffold to wild-type Fc (Figures 4D,E).

### Wild-Type Fc-Fused Protein Monomers

Naturally occurring monomeric cytokines and hormones, such as erythropoietin (Epo), interferon α/β (IFNα/β), and Factor IX (FIX), Fc-fused proteins in the native monomer format, dubbed Fc-fused monomers, showed improved pharmacokinetics and even biological activities, compared to Fc-fused dimers (83). Epo is a glycoprotein hormone drug of ~34 kDa that exerts its effects by binding to the Epo receptor to stimulate red blood cell production (84). Bitonti et al. (85, 86) demonstrated that human IgG1 Fc-fused Epo in monomeric format (Epo-Fc monomer), consisting of a single molecule of Epo conjugated to a wild-type Fc dimer (Figure 4A), exhibited enhanced pharmacokinetic and pharmacodynamic properties, compared to the Epo-Fc dimer, in both cynomolgus monkeys and humans. The inferior activity of the Epo-Fc dimer might be attributed to steric hindrance introduced by two molecules of Epo linked to a dimeric Fc; this could adversely affect the binding to respective receptors (83). To prepare an Epo-Fc monomer, two plasmids encoding FLAG-tagged Epo-Fc at the C-terminus (Epo-Fc-FLAG) and untagged Epo-Fc were first co-transfected into CHO cells. Three protein products were produced by the cells: the Epo-Fc/Epo-Fc homodimer (Epo-Fc dimer), the EpoFc/Epo-Fc-FLAG heterodimer (Epo-Fc monomer), and the Epo-Fc-FLAG/Epo-Fc-FLAG homodimer. The Epo-Fc monomer was purified using a combination of protein A chromatography, size-exclusion chromatography, and subsequently FLAG-affinity chromatography (85, 87).

Similar to the Epo-Fc monomer, Fc-fused monomer formats of IFNβ, IFNα, and FIX exhibited much better biological activity in the monkey models compared to corresponding Fc-fused dimers (83). Intriguingly, in the case of IFNα, the IFNα-Fc monomer showed very similar pharmacokinetics to that of the IFNα-Fc dimer; however, the IFNα-Fc monomer displayed much better *in vivo* efficacy compared to the IFNα-Fc dimer (83). This enhanced efficacy of IFNα-Fc monomer was also dependent on the length of the linker between the IFNα and Fc regions. Longer linker length resulted in increased specific activity of the IFNα-Fc monomer, indicating that there might have been some steric hindrance between IFNα and its receptor. The Fc-fused monomer was then prepared as described previously for the Epo-Fc monomer. Thus, the desired Fc-fused monomer could theoretically be prepared up to only 33% of the total expression yield. Despite many desirable properties of Fc-fused monomers, the development of wild-type Fc-based monomers faces difficulties associated with isolating desired Fc-fused monomers from other format mixtures using robust purification steps, and is also limited by the inherent poor yield of a maximum of 33%.

For Fc-fused monomeric peptides, Kang et al. from Hanmi Pharm (88) developed a glucagon-like peptide-1 (GLP-1)/glucagon (GCG) dual agonist for the potential treatment of obesity and diabetes by activating both GLP-1 and GCG receptors based on LAPScovery™ (Long-Acting Protein/Peptide Discovery) technology. They chemically conjugated GLP-1/GCG peptides to only one arm of human IgG1 aglycosylated Fc fragments, produced from *E. coli* via a flexible polyethylene glycol (PEG) linker (Figure 4B). The GLP-1/GCG-Fc monomer exhibited markedly improved pharmacokinetics and greater *in vivo* biological activity compared to the GLP-1/GCG-Fc dimer (89). However, the preparation of the GLP-1/GCG-Fc monomer was complicated, requiring separate Fc preparation, *in vitro* chemical conjugation of the GLP-1/GCG peptide to the Fc via a PEG linker, and, finally, multi-step purification of the desired product from the reaction mixture (89). Based on LAPScovery technology, aglycosylated Fc-fused monomers for insulin, human growth hormone, and granulocyte-colony stimulating factor were developed, and are now in clinical trials in Europe.^4^

### Wild-Type Fc-Fused Protein Heterodimers

For heterodimeric proteins composed of two different subunits, the Fc-fusion format, achieved by fusion of each subunit independently to separate Fc fragments might be desirable to maintain biological activity, in comparison to the homodimeric form by tandem subunit linkage to wild-type Fc. Follicle-stimulating hormone (FSH) is a non-covalently linked heterodimeric protein consisting of α and β subunits, and its recombinant protein is commonly used for therapy in the treatment of both male and female infertility (90). However, it requires daily injections for several days for up to months due to its short half-life (35 h in humans) (90). To prepare long-acting FSH, Low et al. (91) designed a human IgG1 Fc-fused FSH in two different formats. One is a single chain-linked FSH-Fc (Fc-FSH tandem homodimer) with tandemly linked α-β subunits fused to the N-terminus of the Fc fragment. The other is a heterodimeric FSH-Fc (Fc-FSH heterodimer) with α and β subunits fused separately to the N-termini of each arm of the Fc fragment (Figure 4C). They found that the Fc-FSH heterodimer was significantly more active than the Fc-FSH tandem homodimer, whereas the two Fc-fusion proteins had similar terminal serum half-lives in both rodents and monkeys. It is likely that the architecture of the Fc-FSH heterodimer might present the α and β subunits in a more favorable conformation for bioactivity than that of the Fc-FSH tandem homodimer. To purify Fc-FSH heterodimers, they inserted a 6 × His-tag into the C-terminus of the β subunit-fused Fc fragment only. As a result, whereas the Fc-FSH tandem homodimer was ~90% pure following a single protein A chromatography step, the Fc-FSH heterodimer required additional purification by nickel affinity chromatography to achieve similar purity. However, in addition to multi-step purifications, such preparations of Fc-FSH heterodimers gave lower purification yields (33%) compared to the total expression yield.

### Antibody-Fused Cytokines, Immunocytokines

Cytokines are key players in controlling immune responses during physiological and pathophysiological processes (92). Various cytokines have been clinically approved for diverse immune-related diseases, including interleukin-2 (IL2) and tumor necrosis factor (TNF) as well as INFα for malignant cancer therapy (92, 93). However, the therapeutic efficacy of cytokines is often hampered by poor pharmacokinetics and significant toxicity associated with their systemic administration. To overcome such limitations, antibody–cytokine fusion proteins, the so-called immunocytokines, have been devised in an attempt to enhance antibody-mediated tumor tissue targeting of cytokines, and to increase serum half-lives (93, 94). However, a recent study in mice using an immunocytokine of IL2-fused to tumor antigen-specific IgG in format of IgG-IL2, showed that the biodistribution and pharmacokinetics are entirely governed by the cytokine IL2 moiety, rather than the expected antibody-targeting antigen specificity (95). The IgG-IL2 predominantly remained systemic and was associated with innate immune cells expressing IL2 receptors regardless of antigen specificity or FcγR interactions, whereas the parent IgG antibody was preferentially accumulated in antigen-targeted tumors (95). Rather than the IgG-IL2 immunocytokine, a combination of an anti-tumor antigen IgG antibody and an untargeted Fc-IL2 monomer was approached for synergistic anti-tumor immunotherapy (96).

Furthermore, the biodistribution of immunocytokines was considerably influenced by the ratio of antibody to cytokine. IL12 is a heterodimer (~70 kDa) consisting of heavy (p40) and light (p35) chain subunits, which are covalently linked by disulfide bonds (97). In an attempt to develop IL12-based immunocytokines combined with an L19 scFv that targets the EDB domain of fibronectin, Gafner et al. (98) constructed three different forms of immunocytokines differing in the ratio of antibody to cytokine. They found that the format with 2:1 antibody to cytokine ratio of p40-L19 scFv/L19 scFv-p35, a heterodimeric fusion protein in which the disulfide-linked p35 and p40 subunits of IL12 are fused to L19 scFv, displayed the highest tumor-targeting performance in biodistribution studies. Thus, enhanced therapeutic activity was observed when compared to the other two formats having 1:1 or 2:2 ratio of antibody to cytokine. Similar results were also obtained with IL12 immunocytokines generated with a different F8 scFv antibody (99). With a monomeric cytokine of IL7 (~20 kDa), the same group evaluated the biodistribution of three different formats of IL7-fused F8 scFv immunocytokines differing in antibody to cytokine ratios (100). They found that, as in the case of IL12, the format with a 2:1 antibody to cytokine ratio showed the highest tumor localization compared to the other two formats with 1:1 and 2:2 ratios.

### Heterodimeric Fc-Fused Immunocytokines

The above results suggest that increasing the antibody to cytokine ratio results in improved tumor tissue localization via decreasing cytokine-mediated targeting, which can potentially reduce the dose-related toxicity. IgG-based immunocytokines have been typically generated by fusion of cytokines to the C-terminus of homodimeric Fc fragments of HCs, which present the cytokine as a homodimeric form (31). However, such IgG-based immunocytokines might display poor pharmacokinetics and distribution, as shown with IgG-IL2 formats (95, 101). To generate an IgG-based cytokine monomer format for monomeric (e.g., IL2, IL4, IL7, IL9, IL15, and IL21) and heterodimeric cytokines (e.g., IL12, IL23, IL27, and IL35), a heterodimeric Fc is an attractive scaffold to present cytokines with native structures. Roche (102, 103) generated CEA-targeted IgG-fused IL2 monomeric immunocytokines (CEA-IL2v) by fusion of an engineered IL2 variant (IL2v) to the C-terminus of an antibody with the KiH heterodimeric Fc to obtain a 2:1 antigen-binding valence to cytokine ratio (Figure 4D). Similarly, they also developed the fibroblast activation protein (FAP)-directed IgG-IL2v monomeric immunocytokine (FAP-IL2v) (103, 104). Both CEA-IL2v (NCT02004106, NCT02350673) and FAP-IL2v (NCT02627274) are now being evaluated in Phase 1 clinical trials.

## Conclusion and Prospects

In summary, immunoglobulin Fc heterodimer technology is very useful in generation of bsAbs with various antigen specificities and binding valencies in full-length IgG and IgG-like formats, while retaining many favorable properties of natural IgG antibodies. As of July 2016, more than seven heterodimeric Fc-based IgG-format bsAbs are now being evaluated in clinical trials (Table 2). Nonetheless, there are some issues to be considered for heterodimeric Fc-based bsAbs. All of the heterodimeric Fc-based bsAbs require assembly with three or four separate chains, which often hampers cell line development for high yield expression. Since each heterodimeric Fc chain has different mutations in each CH3 domain, the expression level and stability are somewhat different from each other, often requiring optimizing processes to identify the best pair between heterodimeric Fc chain and fusion partner (35). Furthermore, since the so far developed Fc heterodimers do not reach a 100% heterodimerization yield, the downstream process to ensure high bsAb purity, to meet clinical needs, should be considered from the initial stage of the heterodimeric Fc-based bsAb design.

In addition to bsAbs, heterodimeric Fc technology is now emerging as a promising scaffold for the generation of Fc-fused proteins and cytokines in monomeric or heterodimeric formats. The generation of Fc-fused monomeric proteins with wild-type Fc fragments is not any more desirable because it requires additional artificial sequences, such as extra tags for selective affinity chromatography and also multiple purification steps with limited purification yields (maximum 33%). Fusion to heterodimeric Fc fragments can present the fusion partner in its natural monomeric form for monomeric proteins and peptides as well as natural heterodimeric forms for heterodimeric proteins, rather than the artificial homodimeric form for wild-type Fc fragments (Figure 4E). This heterodimeric Fc capability will facilitate the development of the next generation of Fc-fused proteins that maintain full function of the fusion partner, while retaining the Fc-mediated extended half-life and immune effector functions. The reduced binding valency might further help to reduce target receptor-mediated clearance. Particularly, in tumor-targeting IgG-based immunocytokines, the heterodimeric Fc-fused cytokines, in their natural forms, improve tumor tissue accumulation by reducing associations with immune cells, and thereby minimizing systemic toxicity. This facilitates their development as therapeutic agents. Some heterodimeric Fc-based IgG-fused immunocytokines are now in clinical trials and many more are expected to be developed.

## Author Contributions

JHH, JEK, and YSK designed the review article topic, surveyed relevant literatures and patents, and analyzed the data; YSK designed and supervised the review article; and JHH and YSK wrote the manuscript with input from all the other authors.

## Conflict of Interest Statement

The authors declare that the research was conducted in the absence of any commercial or financial relationships that could be construed as a potential conflict of interest.

## Footnotes

^1^http://xencor.com/

^2^http://www.merus.nl/

^3^http://www.zymeworks.com/

^4^http://www.hanmipharm.com/
