# Corrigendum: Immunoglobulin Fc Heterodimer Platform Technology: From Design to Applications in Therapeutic Antibodies and Proteins

**DOI:** 10.3389/fimmu.2017.01582

**Published:** 2017-11-13

**Authors:** Ji-Hee Ha, Jung-Eun Kim, Yong-Sung Kim

**Affiliations:** ^1^Department of Molecular Science and Technology, Ajou University, Suwon, South Korea; ^2^Department of Applied Chemistry and Biological Engineering, College of Engineering, Ajou University, Suwon, South Korea

**Keywords:** bispecific antibody, Fc engineering, heterodimeric Fc, Fc-fusion proteins, immunocytokines, antibody engineering

In the original article, there was an error [wrong description on LY3164530 (Eli Lilly) antibody in the last paragraph of page 9 of original article].

A correction has been made to section “HETERODIMERIC Fc-BASED ANTIBODIES IN DIVERSE FORMATS”, subsection “Intact IgG Formats with Correct LC Association”, sixth Paragraph (line 8–12 of the sixth paragraph) (In the last paragraph of page 9 of original article):

An alternative approach for enforcing correct HC_VH-CH1_–LC association includes introduction of a set of mutations at the heterodimeric VL–CL and VH–CH1 interface (18, 66, 67), similar to modification of the CH3 interface for the heterodimeric Fc design. In an ortho-Fab IgG approach (18), structure-based regional design introduced complementary mutations at the LC and HC_VH-CH1_ interface in only one Fab, without any changes being made to the other Fab (Figure 3). Zymeworks is currently developing intact IgG-format bsAbs generated by the combination of ortho-Fab IgG and ZW1 Fc technologies (http://www.zymeworks.com/).

The authors apologize for this error and state that this does not change the scientific conclusions of the article in any way.

## Conflict of Interest Statement

The authors declare that the research was conducted in the absence of any commercial or financial relationships that could be construed as a potential conflict of interest.

